# Rate of Change of Rapid Shallow Breathing Index and Extubation Outcome in Mechanically Ventilated Patients

**DOI:** 10.1155/2023/9141441

**Published:** 2023-09-26

**Authors:** Manjush Karthika, Farhan A. Al Enezi, Lalitha V. Pillai, Yaseen M. Arabi

**Affiliations:** ^1^Faculty of Medical and Health Sciences, Liwa College, Abu Dhabi, UAE; ^2^Faculty of Medical and Health Sciences, Symbiosis Centre for Research and Innovation, Symbiosis International University, Pune, India; ^3^Intensive Care Department, King Abdulaziz Medical City, King Saud Bin Abdulaziz University of Health Sciences, King Abdullah International Medical Research Center, Riyadh, Saudi Arabia; ^4^Department of Critical Care Medicine, Aundh Institute of Medical Sciences, Pune, India

## Abstract

**Background:**

Rapid shallow breathing index (RSBI) has been widely used as a predictor of extubation outcome in mechanically ventilated patients. We hypothesize that the rate of change of RSBI between the beginning and end of a 120-minute spontaneous breathing trial (SBT) could be a better predictor of extubation outcome than a single RSBI measured at the end of SBT in mechanically ventilated patients. *Methodology*. In this prospective observational study, we enrolled 193 patients who met the inclusion criteria, of whom 33 patients were unable to tolerate a 120-minute SBT and were excluded from the study. The study population consisted of 160 patients, categorized into three subgroups: patients with normal lung (no reported history of respiratory diseases), patients with airway disease, and patients with parenchymal disease who completed 120 minutes of SBT on low levels of pressure support ventilation. RSBI was obtained from the ventilator display at the 5^th^ and the 120^th^ minutes of SBT. The rate of change of RSBI (RSBI 5–120) was calculated as (RSBI 2-RSBI 1)/RSBI 1 × 100. Receiver-operating characteristic (ROC) curves were plotted for RSBI 5–120 and RSBI 120 in all patients and among the three subgroups (normal group, airway group, and parenchymal group) to compare the superiority of their best thresholds in predicting extubation failure.

**Results:**

The RSBI 5–120 threshold for extubation failure in the entire patient group was 23% with an overall accuracy of 88% (AUC = 0.933, sensitivity = 91%, and specificity = 86%) and the threshold of RSBI 120 for extubation failure in the entire patient group was 70 breaths/min/L with an overall accuracy of 82% (AUC = 0.899, sensitivity = 85%, and specificity = 81%). In patients in the normal lung group, the threshold of RSBI 5–120 was 22%, with an overall accuracy of 89% (AUC = 0.892, sensitivity = 87.5%, and specificity = 90%), and the RSBI 120 threshold was 70 breaths/min/L, with an overall accuracy of 89% (AUC = 0.956, sensitivity = 88%, and specificity = 90%). The RSBI 5–120 threshold in patients with airway disease was 25% with an accuracy of 86% (AUC = 0.892, sensitivity = 85%, and specificity = 86%) and the threshold of RSBI 120 was 73 breaths/min/L with an accuracy of 83% (AUC = 0.874, sensitivity = 85%, and specificity = 82%). In patients in the parenchymal disease group, the threshold of RSBI 5–120 was 24%, with an accuracy of 90% (AUC = 0.966, sensitivity = 92%, and specificity = 89%) and RSBI 120 threshold was 71 breaths/min/L, which was 88% accurate (AUC = 0.893, sensitivity = 85%, and specificity = 89%).

**Conclusion:**

The rate of change of RSBI between the 5^th^ and 120^th^ minutes was moderately more accurate than the single value of RSBI measured at the 120^th^ minute in predicting extubation outcome.

## 1. Background

Prediction of successful liberation from mechanical ventilation (MV) is important in clinical decision-making in intensive care units (ICUs) [1, 2]. Premature liberation and undue delays in MV weaning are associated with undesirable patient outcomes and increased length of stay in the ICU [3, 4]. Premature MV discontinuation places additional stress on the respiratory and cardiovascular systems. At the same time, an undue delay may lead to diaphragmatic atrophy [5], venous thromboembolism, delirium, pneumonia, and factors associated with increased mortality and morbidity [6–8]. Delaying MV discontinuation needs to be balanced against possible premature discontinuation; medical patients spend as much as 42% of their total MV time during the weaning process [9].

Also, in real-life practice, clinical judgment alone may not always be sufficient to accurately predict weaning outcomes [10, 11] which warrants the search for better indices. Certain physiological measurements such as maximum inspiratory pressure (PImax) that represents respiratory muscle strength [12], or certain weaning indices that factor only one function have shown to be less accurate than integrated indices such as the integrative weaning index (IWI) that combine respiratory mechanics, oxygenation, and breathing pattern, or the compliance, rate, oxygenation, and pressure (CROP) index and the dynamic compliance, oxygenation, rate, and effort (CORE) index which is a modification of the CROP index [13–15]. However, none of these indices have proven to be perfect. Since its description by Yang and Tobin in 1991 [16], the rapid shallow breathing index (RSBI) which is a ratio of respiratory rate (RR) to tidal volume (VT) has been used much more extensively owing to its relative simplicity and accurate threshold values of >105 breaths/min/L predictive of weaning failure while RSBI <105 breaths/min/L is predictive of weaning success [16].

Various researchers subsequently explored modifications such as serial RSBI measurements and the rate of change of RSBI during spontaneous breathing trials (SBT). Segal et al. in 2010 defined RSBI rate as the rate of change of RSBI in serial measurements [17]. The authors also concluded that a percent change in RSBI during SBT predicted successful extubation to a greater extent than a single RSBI measurement in a heterogeneous patient population. However, the change in the percentage of RSBI in disease-specific groups such as airway or parenchymal disorders has not been studied. In this study, we hypothesize that the rate of change of RSBI between the beginning and end of a 120-minute spontaneous breathing trial (SBT) could be a better predictor of extubation outcome than a single RSBI measured at the end of SBT in mechanically ventilated patients. We also assess the relationship between the rate of change of RSBI during SBT and extubation outcomes in ventilated patients with airway disease, parenchymal disease, and no reported respiratory diseases.

## 2. Methodology

This prospective, observational study was conducted in association with three institutions in Saudi Arabia (King Abdulaziz Medical City, Riyadh) and India (AIMS Hospital and Symbiosis International University, Pune). Ethical approval was obtained from the respective Institutional Ethical Boards. Informed consent was waived due to the observational nature of this study. We included patients with respiratory failure from airway diseases (asthma and chronic obstructive pulmonary diseases), patients with respiratory failure from parenchymal diseases (pneumonia and other lung tissue diseases), and patients with no known history of, or documented respiratory system disorders but presented with respiratory failure due to other causes (e.g., secondary to trauma or postsurgical phase). We included patients who were ≥18 years of age, received invasive ventilation for >24 hours, were intubated with an endotracheal tube (ET tube) of ≥7.5 mm internal diameter, conscious and neurologically stable, in their first weaning attempt (two hours SBT), have partial or complete recovery from respiratory failure and clinical signs of improvement from the precipitating cause, have stable hemodynamics with minimal/without the need for vasoactive drugs, and have no other comorbidities related to other organ failure. The key exclusion criteria were ventilator duration <24 hours, tracheostomy, second weaning attempt, accidental extubation during SBT, ET tube change because of obstruction during SBT, discharge against medical advice during the study, patients with fibrotic lung diseases, and neuromuscular diseases. The patients were categorized as normal group (patients with no reported history of respiratory diseases), airway group (patients with underlying airway diseases such as asthma or chronic obstructive pulmonary diseases), and parenchymal group (patients with parenchymal involvement such as pneumonia or pulmonary edema or other lung tissue diseases).

### 2.1. General Respiratory Management

Recruited patients were managed as per local standards by ICU physicians and respiratory therapists (RTs). They were monitored as per local practices for various clinical factors such as oxygenation status, ventilation status, airway adequacy, airway and gag reflexes, hemoglobin (Hb), cardiac and hemodynamic stability, and neuromuscular status. Patients were studied in a semirecumbent position with the head of the bed elevated to an angle of 30–45 degrees for the ease of breathing and comfort during transition [18]. ET and oral suctioning were performed before SBT, with three-minute pre- and postoxygenation. As per standard practices, ET tube cuff pressure was inflated to 25 cm H_2_O to prevent leak and aspiration [19, 20].

Before use, the ventilators (Drager, Maquet, Puritan Bennett, and GE) and flow sensors were calibrated on a periodic maintenance basis and were checked for leaks. Ventilators had apnea backup, which was set appropriately. Before SBT, upper and lower limits of tidal volume (VT), respiratory rate (RR), exhaled minute ventilation (VE), and airway pressure alarms were set appropriately. Arterial blood gas (ABG) measurements were performed in elective patients based on physicians' decisions, and hence ABG variables were not included in the study.

### 2.2. SBT

The time of SBT commencement was noted. During the weaning process, ventilation mode was switched to “spontaneous” mode, i.e., PSV with a pressure support level of 5–7 cm H_2_O, positive end-expiratory pressure (PEEP) of 5 cm H_2_O and fraction of inspired oxygen (FiO_2_) not exceeding 40%. We started assessing the RSBI at the fifth minute, considering the importance of transition and neuro-ventilatory efficiency and observing for any harmful effects of respiratory muscle fatigue, as they occur early in failing SBTs [21–23]. Patients who did not tolerate the SBT during the first five minutes with apparent signs of distress decreased respiratory efforts, hypoxic signs, restlessness, and hemodynamic derangements were switched back to the previous controlled ventilation mode. The trial was continued for those patients who tolerated the initial five minutes of SBT, and the RSBI was obtained from the ventilator display at the fifth minute. For the patients who could not tolerate SBT due to any eventful subjective or objective presentation during the 120 minutes, pressure support levels were increased or were switched back to the previous mode of controlled ventilation, and they were rested for the next 24 hours or as per the decisions of the ICU or primary physicians. Glasgow coma scale (GCS), systolic blood pressure (SBP), heart rate (HR), RR, and VT were charted every 30 minutes from onset to 120 minutes of SBT. At the end of 120 minutes, patients who were alert and arousable, had stable vitals and hemodynamics, followed commands, had intact cough and gag reflex, and had minimal amount of secretions were considered to be fit for extubation after being assessed for subglottic edema by cuff-leak test. The patients who were not extubated were categorized under weaning failure. Although many of the factors of weaning failure, such as hemodynamic, neurological, and respiratory causes were noted, it was also based on the discretion of the ICU physician if a poor outcome was anticipated. Hence, the causes of weaning failure were not included in the analysis.

Successfully extubated patients were supported with supplementary oxygen, nebulized bronchodilators, adrenaline as needed, active coughing and huffing, chest physiotherapy, deep breathing exercises and incentive spirometry, and remained in the ICU for the next 24 hours. During these 24 hours postextubation, patients were closely observed for any derangement in respiratory mechanics, neurological, oxygenation, ventilatory, cardiac, hemodynamic or circulatory status. Oxygen supplementation and noninvasive ventilator support were provided to extubated patients per the requirement. During the 24 hours postextubation, patients with eventful presentation related to respiratory, neurological, cardiac status, or hemodynamic derangements were reintubated and reinstituted on mechanical ventilation.

### 2.3. Measurements and Definitions

Patients were classified into three outcome groups based on their status up to 24 hours postextubation: (1) Weaning failure: included patients who were not extubated after two hours of SBT for various clinical reasons at the primary physician's discretion. (2) Extubation failure: included patients who required invasive airway within 24 hours of extubation. (3) Extubation success: included patients who did not require ET reintubation within 24 hours postextubation. 24 hours was used since all the patients would remain in ICU care for at least 24 hours following extubation. Also, the fact that one in five patients may require reintubation during hospital stay, with half of these patients requiring reintubation within the first 24 hours, with a median time of 22 hours [24, 25], was taken into consideration.

RSBI obtained during the SBT at two intervals (5^th^ and 120^th^ minute) was used to calculate the RSBI rate. We obtained the RSBI values from the ventilator display. It has been proven that RSBI calculated from values obtained by direct ventilometry and the one obtained through the display of the mechanical ventilator are highly correlated [26]. RSBI rate was calculated by using the following formula: (RSBI 2–RSBI 1)/RSBI 1 × 100, where RSBI 1 is the initial RSBI value (fifth minute) and RSBI 2 is the second value measured at the end of 120 minutes. RSBI rate is the rate of change of RSBI between two intervals and is denoted as RSBI 5–120, expressed in percentage.

### 2.4. Statistical Analysis

Statistical Package for the Social Sciences (SPSS) software, version 21 (IBM® SPSS Statistics 21) was used to analyze the coded data. Continuous data are presented as mean ± SD unless otherwise indicated. Descriptive statistics were used to explain demographic variables. Demographic and clinical variables were compared for the groups using analysis of variance (ANOVA) with post hoc pairwise testing utilizing Tukey's post hoc honest significant difference test (Tukey's HSD). Multivariate logistic regression using forward stepwise selection analysis was performed to analyze the association of general and specific variables such as age, gender, ET tube size, Hb, GCS, SBP, HR, RR, and VT in predicting extubation failure. Using the identified variables, we generated a final logistic regression model to predict the probability of extubation failure. Receiver-operating characteristic (ROC) curves were plotted to compare the optimal thresholds of RSBI 5–120 and RSBI 120 that can predict extubation failure in the four study groups. Sensitivity, specificity, positive predictive value (PPV), negative predictive value (NPV), positive likelihood ratio (PLR), negative likelihood ratio (NLR), and overall accuracy were identified for both thresholds in each group.

## 3. Results

Of the 193 patients, 33 were excluded (SBT intolerance) from the analysis due to early clinical intolerance during 120 minutes of SBT, precluding measurement of RSBI at the 120^th^ minute. 12 out of 33 were excluded within the first five minutes before the 1^st^ RSBI value. The remaining 160 patients (normal lung = 49, airway group = 55, and parenchymal group = 56) who completed two hours of SBT were included for final analysis.

Out of the 33 patients who were excluded as early intolerance, 24 (73%) had acute respiratory, 8 (24%) had hemodynamic, and 1 (3%) had neurological deterioration. 47 patients failed weaning during 120 minutes of SBT and 34 (74%) of them presented respiratory issues, 10 (22%) had hemodynamic problems, and the remaining 2 (4%) patients had neurological deterioration. 34 patients failed the extubation, in which 25 (74%) patients had respiratory deterioration, 7 (21%) patients had hemodynamic compromise, and 2 (6%) of them had neurological worsening. Most of the respiratory worsening was presented as tachypnea and respiratory distress. Hemodynamic issues involved hypo/hypertension, brady/tachy arrhythmias, and prearrest scenarios. Neurological worsening was presented as decreased level of consciousness and altered mentation.

**Figure 1:**
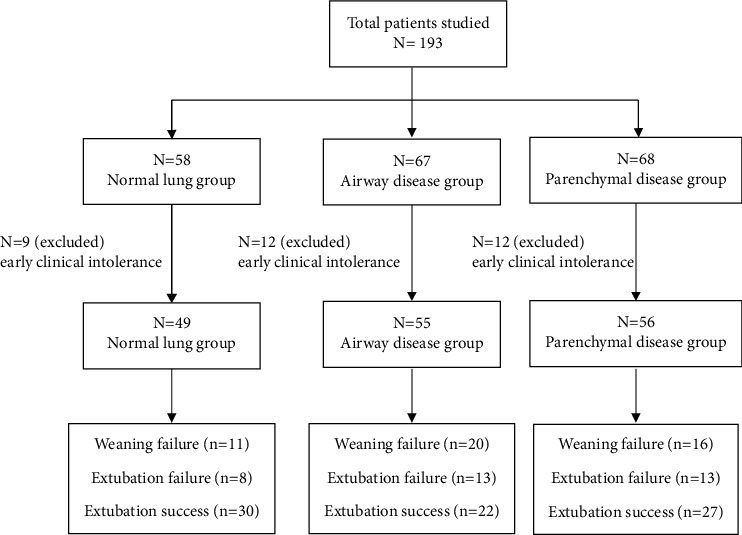
Overall patient distribution.

The overall distribution of the patients is depicted in Figure 1.

### 3.1. Whole Patient Group

Of the 160 patients, 99 were males (62%) and 61 were females (38%). The average age was 58 ± 15.55 years. Forty-seven patients (29.4%) failed weaning, 34 (21.3%) failed extubation, and the remaining 79 patients (49.4%) had successful extubation. The mean RSBI 120 was 83.57 ± 9.72, 73.41 ± 3.64, and 63.11 ± 6.94 for the weaning failure, extubation failure, and extubation success groups, respectively. Tukey's HSD confirmed a significant difference in RSBI 120 between the three outcome groups (*P* ≤ 0.001). The mean RSBI 5–120 was 38.45 ± 10.36, 32.41 ± 8.35, and 21.18 ± 3.66 for the weaning failure, extubation failure, and extubation success group, respectively. Tukey's HSD confirmed a significant difference in RSBI 5–120 between the three outcome groups (*P* ≤ 0.001). Multivariate logistic regression followed by forward stepwise selection identified GCS, HR, RR, and TV as independent predictors of extubation failure (*P* = 0.037, 0.017, 0.001, and 0.000, respectively). A final multivariate logistic regression model confirmed the significance of the identified variables such as GCS, HR, RR, and VT in predicting extubation failure (Table 1). This model correctly identified 96% of the extubation failures and 79% of the extubation successes with an overall prediction accuracy of 91%.

**Figure 2:**
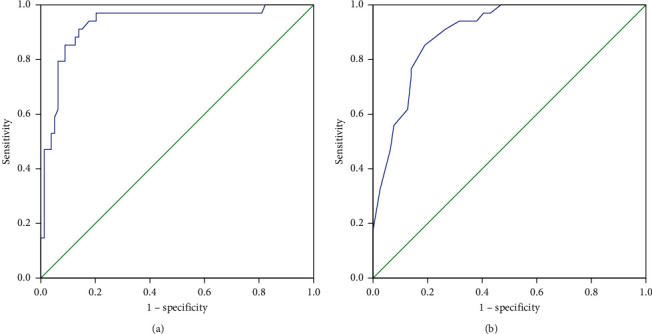
Receiver-operating characteristic curve comparing the predictive thresholds in the whole patient group: (a) RSBI 5-120 (AUC = 0.933) and (b) RSBI 120 (AUC = 0.899).

ROC curves were plotted to compare the performance of RSBI 5–120 and RSBI 120 in predicting extubation failure and identifying the optimal thresholds (Figure 2). The ROC curve plotted for RSBI 5–120 in the whole patient group yielded an AUC of 0.933. A rate of change of 23% was determined as the optimal threshold for predicting extubation failure in RSBI 5–120 (sensitivity = 91%, specificity = 86%, PLR = 6.56, NLR = 0.10, PPV = 74%, NPV = 96%, and the overall accuracy of threshold = 88%).

The ROC curve plotted for RSBI 120 in the whole patient group yielded an AUC of 0.899. A threshold of 70 was identified as optimal for predicting extubation failure in the entire patients for the RSBI 120 variable (sensitivity = 85%, specificity = 81%, PLR = 4.49, NLR = 0.18, PPV = 66%, NPV = 93%, and the overall accuracy of threshold = 82%).  Table 2 summarizes the demographic and clinical characteristics of the 160 patients based on the study's outcome.

### 3.2. Normal Group

Of the 49 patients, 33 were males (67%) and 16 were females (33%). The average age was 56 ± 17.45 years. 11 patients (22.4%) failed weaning, 8 (16.3%) failed extubation, and the remaining 30 patients (61.2%) were categorized under successful extubation. The mean RSBI 120 was 80.18 ± 8.21, 75.25 ± 3.28, and 60.89 ± 8.17 for the weaning failure, extubation failure, and extubation success group, respectively (*P* < 0.05). Tukey's HSD confirmed a significant difference in RSBI 120 between the three outcome groups (*P*=0.003), a significant difference was not observed between weaning failure and extubation failure (*P*=0.181). The mean RSBI 5–120 was 36.4 ± 9.34, 29.25 ± 6.66, and 20.55 ± 1.64 for the weaning failure, extubation failure, and extubation success group, respectively (*P*=0.005). Tukey's HSD confirmed a significant difference in RSBI 5–120 between the three outcome groups (*P*=0.014). The differences in clinical characteristics across the groups are presented in the supplemental material. Multivariate logistic regression followed by forward stepwise selection identified GCS, RR, and TV as independent predictors of extubation failure (*P*=0.037, 0.017, 0.001, and ≤0.001, respectively). A final multivariate logistic regression model confirmed the significance of the identified variables such as GCS, RR and VT in predicting extubation failure (Table 1). This model correctly identified 95% of the extubation failures and 97% of extubation successes with an overall prediction accuracy of 96%.

**Figure 3:**
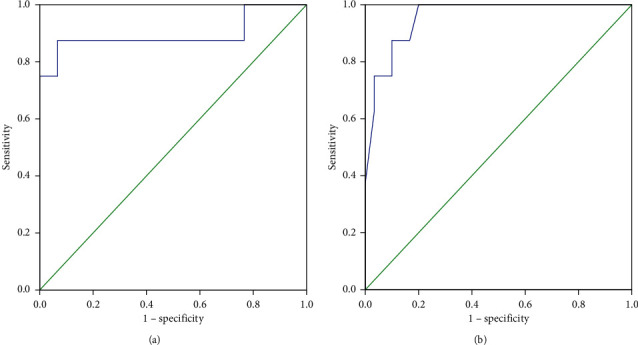
Receiver-operating characteristic curve comparing the predictive thresholds in the normal lung group: (a) RSBI 5-120 (AUC = 0.892) and (b) RSBI 120 (AUC = 0.956).

The ROC curve plotted for RSBI 5–120 in the normal patient group yielded an AUC of 0.892. A threshold of 22% was identified as the rate of change of RSBI 5–120 to predict extubation failure (sensitivity = 87.5%, specificity = 90%, PLR = 8.75, NLR = 0.14, PPV = 70%, NPV = 96%, and the overall accuracy of threshold = 89%). An AUC of 0.956 was yielded from the ROC plotted for RSBI 120 in this group. A threshold of 70 was identified as optimal for RSBI 120 in predicting extubation failure in normal lung patients (sensitivity = 88%, specificity = 90%, PLR = 8.75, NLR = 0.14, PPV = 70%, NPV = 96%, and the overall accuracy of threshold = 89%) (Figure 3).

### 3.3. Airway Group

Out of the 55 patients with airway disease, 31 were males (56%) and 24 were females (44%). The average age was 62 ± 8.71 years. Out of the 55 patients, 20 patients (36.4%) failed weaning, 13 (23.6%) failed extubation, and the remaining 22 (40.0%) were categorized under successful extubation. The mean RSBI 120 was 86.2 ± 11.51, 73.69 ± 3.33, and 64.86 ± 6.76 for the weaning failure, extubation failure, and extubation success group, respectively (*P* < 0.05). Tukey's HSD confirmed that the individual outcome group differed significantly from the others in RSBI 120 (*P* ≤ 0.001). The mean of RSBI 5–120 was 39.23 ± 12.09, 32.67 ± 8.21, and 23.05 ± 5.6 for the weaning failure, extubation failure, and extubation success group, respectively (*P* < 0.05). Tukey's HSD confirmed a significant difference between the extubation success group versus extubation failure and weaning failure (*P* ≤ 0.001 and 0.010), while no significant difference was observed between extubation failure and weaning failure groups (*P*=0.114). The differences in clinical characteristics across the groups are presented in the supplemental material. Forward stepwise selection identified RR and VT as independent predictors of extubation failure (*P*=0.015). The final multivariate logistic regression confirmed the significance of identified variables, such as RR and VT, in predicting extubation failure (Table 1). This model correctly identified 94% of the extubation failures and 77% of the extubation successes, with an overall prediction accuracy of 87%.

**Figure 4:**
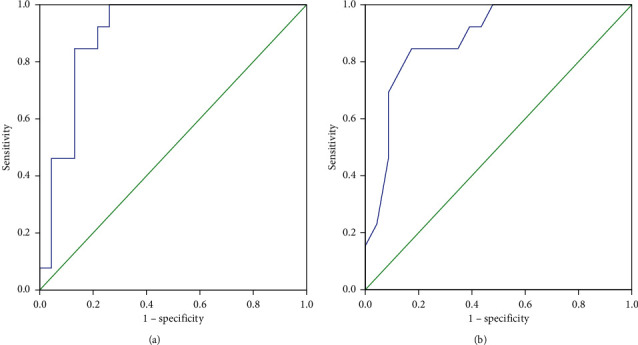
Receiver-operating characteristic curve comparing the predictive thresholds in the airway disease group: (a) RSBI 5-120 (AUC = 0.892) and (b) RSBI 120 (AUC = 0.874).

The ROC curve plotted for RSBI 5–120 in the airway group yielded an AUC of 0.892. The curve identified a rate of change of 25% as the optimal threshold for predicting extubation failure (sensitivity = 85%, specificity = 86%, PLR = 6.22, NLR = 0.18, PPV = 79%, NPV = 90%, and the overall accuracy of threshold = 86%). The ROC curve plotted for RSBI 120 in the airway group yielded an AUC of 0.874. A threshold of 73 was identified as optimal for RSBI 120 in predicting extubation failure in patients with airway diseases (sensitivity = 85%, specificity = 82%, PLR = 4.65, NLR = 0.19, PPV = 73%, NPV = 90%, and the overall accuracy of threshold = 83%) (Figure 4).

### 3.4. Parenchymal Group

Of the 56 patients with the parenchymal disease, 35 were males (67%) and 21 were females (33%). The average age was 57 ± 18.55 years. Out of the 49 patients, 16 patients (28.6%) failed weaning, 13 (23.2%) failed extubation and the remaining 27 (48.2%) were categorized under successful extubation. The mean RSBI 120 was 82.63 ± 7.62, 72 ± 3.83, and 63.83 ± 5.41 for the weaning failure, extubation failure, and extubation success group, respectively (*P* < 0.05). Tukey's HSD confirmed that the individual outcome group was significantly different from each other in RSBI 120 variable (*P*=0.001 and ≤0.001). The mean of RSBI 5–120 was 38.9 ± 9.04, 34.09 ± 9.41, and 20.35 ± 2.76 for the weaning failure, extubation failure, and extubation success group, respectively (*P* < 0.05). Tukey's HSD confirmed a significant difference between extubation success versus weaning failure and extubation failure for the variable RSBI 5–120 (*P* ≤ 0.001). The differences in clinical characteristics across the groups are presented in the supplemental material. The forward stepwise selection approach identified GCS and VT as independent predictors of extubation failure (*P*=0.006 and 0.002). The final multivariate logistic regression model confirmed the significance of identified variables such as GCS and VT in predicting extubation failure (Table 1). This model correctly identified 90% of the extubation failures and 89% of the extubation successes, with an overall prediction accuracy of 89%.

**Figure 5:**
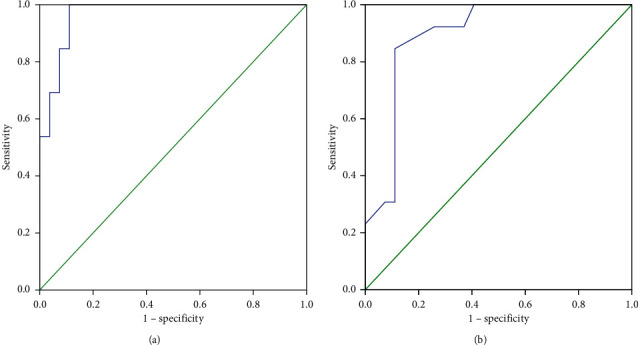
Receiver-operating characteristic curve comparing the predictive thresholds in the parenchymal disease group: (a) RSBI 5-120 (AUC = 0.966) and (b) RSBI 120 (AUC = 0.893).

The ROC curve plotted for RSBI 5–120 in the parenchymal group yielded an AUC of 0.966. The plotted curve determined a rate of change of 24% as the optimal threshold for predicting extubation failure (sensitivity = 92%, specificity = 89%, PLR = 8.32, NLR = 0.09, PPV = 80%, NPV = 96%, and the overall accuracy of threshold = 90%). The ROC curve plotted for RSBI 120 in the parenchymal group yielded an AUC of 0.893. A threshold of 71 was identified as optimal for RSBI 120 in predicting extubation failure (sensitivity = 85%, specificity = 89%, PLR = 7.62, NLR = 0.17, PPV = 79%, NPV = 92%, and the overall accuracy of threshold = 88%) (Figure 5).

## 4. Discussion

Our study assessed the utility of the rate of change of RSBI between the 5^th^ and 120^th^ minutes of SBT as a better predictor of extubation outcome than a single value of RSBI measured at the end of the SBT in the heterogeneous group of patients and disease-specific patient groups. It was identified that the rate of change of RSBI is moderately more accurate in predicting extubation failure than a single value RSBI measured at the end of the SBT, suggesting its usefulness during the weaning phase to assess extubation readiness.

Literature suggests that respiratory muscle weakness and fatigue, particularly associated with the increased respiratory workload, may result in a rapid and shallow breathing pattern [27] which makes it important to consider RSBI as a predictor of weaning outcome during SBTs in a disease-specific patient population.

Our aim was to compare the performance of RSBI 5–120 (rate of change of RSBI between the 5th and 120^th^ minute) and RSBI 120 (single RSBI determinant at the 120^th^ minute). We used PSV to wean the patients, and RSBI values were obtained from the ventilator, considering the diverse respiratory mechanics of the patients, and minimal stress to the patients, as reported by Shingala et al. [28]. Shingala et al. compared RSBI on PSV and RSBI on spontaneous breathing with T-piece and confirmed that RSBI on PSV is a better predictor for extubation, easier to obtain, and causes lesser stress to the patients. Several modifications, such as serial measurements and the rate of change of RSBI, have been suggested to improve its predictive value further [29]. The concept of serial RSBI developed from the observation that the breathing pattern in some patients may be stable at the initiation of SBT but deteriorate later. This deterioration is ascribed to poor respiratory muscle endurance or worsened respiratory mechanics that may not be present at the initiation of SBT [2]. Hence, researchers focused on the serial assessment of RSBI at various intervals. Chatila et al. reported that RSBI measured at 30 min of an SBT was a better predictor of weaning outcome than RSBI at the start of weaning initiation [30]. Agreeing with Chatila et al. and considering the importance of breathing transition, harmful effects of respiratory muscle fatigue as seen in failing SBTs, and neuro-ventilatory efficiency to predict extubation outcome [21–23], we gave a window period of five minutes to obtain the first measurement of RSBI. And we observed that 12 out of 193 patients had an early clinical intolerance during the initial fifth minute of the trial. The causes of this early intolerance were multifactorial, including signs and symptoms of respiratory distress, decreased respiratory efforts, hypoxia, restlessness, and hemodynamic derangements. Compared to the 12 intolerant patients in the initial five minutes, we found that 21 more patients were not able to complete SBT in the next 120 minutes, reiterating the need for various windows of assessment before 120 minutes of SBT, as suggested by Krieger et al. [31]. Another study about serial RSBI measurement and weaning outcome in critically ill patients concluded the superiority of RSBI measured after SBT compared to that measured at the beginning [32].

Many studies have concluded that RSBI thresholds much lower than 105 breaths/min/L better predict weaning or extubation outcome. In a meta-analysis of 65 observational studies, the authors reported a value of <65 breaths/min/L as the optimum cut-off [33]. Our analysis also yielded a lower RSBI threshold in disease-specific groups compared to the original RSBI threshold proposed by Yang and Tobin [2]. Our findings of a lower RSBI threshold agree with previously published studies [34–37]. El-Khatib et al. [34] reported a mean RSBI of 46 ± 8 breaths/min/L, and all patients had RSBI less than 105 breaths/min/L, while Zhang and Qin [35] concluded that an RSBI value of 75 breaths/min/L in PSV was found to be more accurate for predicting successful weaning. Patel et al. [37] reported median and interquartile ranges of single determinant RSBI as 71 (52–88) breaths/min/L in patients on CPAP mode with a PEEP of 5 cm H_2_O during SBT. Danaga et al. [36] reported that the >105 breaths/min/L RSBI cut-off value predicted only 20% of the cases of extubation failure, whereas a threshold of 76.5 breaths/min/L provided substantial improvement in sensitivity, with an acceptable loss of specificity. We assume the difference in our groups' RSBI thresholds is because of their underlying lung and airway conditions.

With continuous evolution in the RSBI techniques, Segal et al. proposed the rate of change in RSBI in serial measurement [17]. Segal et al. conducted serial RSBI measurements during SBT. They hypothesized the rate of change in RSBI as a successful predictor of weaning, considering the dynamic nature of the respiratory failure. In this prospective cohort of 30 patients, after two hours of SBT, the authors identified a threshold of <20% as a predictor of successful weaning. In their subsequent study, the authors concluded that the percent change of RSBI during an SBT is a better predictor of successful extubation than a single determination of RSBI [38]. 63 out of 72 patients were successfully extubated by using the RSBI rate threshold of 20% as the predictor. However, this project included a heterogeneous disease population from medical surgical and cardiac care units without subcategories as per the underlying respiratory mechanics, as seen in airway diseases and parenchymal disorders. In our study, we assessed this concept in disease-specific groups. When we analyzed the entire patients, we identified a rate of change of more than 23% as the ideal threshold that predicts extubation failure, with a sensitivity of 91%, specificity of 86%, and overall accuracy of 88%. Regarding a single determinant RSBI, we identified a threshold of more than 70 breaths/min/L of RSBI at the end of SBT, in predicting extubation failure in this heterogeneous group with a sensitivity of 85%, specificity of 81%, and overall accuracy of 82%. The overall accuracy of RSBI 5–120 in each outcome group was marginally superior compared to the respective RSBI 120 thresholds.

## 5. Conclusion

In conclusion, this study shows that the rate of change of RSBI measured at the beginning and the end of the SBT was moderately more accurate than a single determinant of RSBI measured at the end of the SBT in predicting extubation outcome in a heterogenous group of ventilated patients, specifically in airway and parenchymal disease groups. Because respiratory failure is dynamic in process, measuring RSBI rate at various intervals from the beginning of SBT may reflect a better accuracy of respiratory endurance before extubation, thereby reducing untoward events such as reintubation and related complications.

## Figures and Tables

**Table 1:** Clinical parameters across the outcome groups.

Parameters	
Normal groups	
GCS, HR, RR, VT, and Hb	Significantly different between all three outcome groups
GCS and RR	ES is significantly different vs. EF and WF
GCS and RR	No significant difference between WF and EF
HR and VT	Significant difference between WF vs. ES
HR and VT	No significant difference between EF vs. WF and ES
Hb	Significant difference for WF vs. EF and ES
Hb	No significant difference between ES vs. EF
Airway group	
GCS, HR, RR, and VT	Significantly different between all three outcome groups
GCS and RR	ES is significantly different vs EF and WF
GCS and RR	No significant difference between WF and EF
HR	Significant difference for WF vs. EF and ES
HR	No significant difference between EF and ES
VT	Significant difference for all three outcome groups
Parenchymal group	
GCS, RR, VT, and Hb	Significantly different between all three outcome groups
GCS	ES is significantly different vs. EF and WF
GCS	No significant difference between WF and EF
RR and VT	Significant difference for ES vs. WF and EF
RR and VT	No significant difference between WF and EF
Hb	No significant difference between, ES and WF
Hb	No significant difference between EF and WF

EF, extubation failure; ES, extubation success; GCS, Glasgow coma scale; Hb, hemoglobin; HR, heart rate; RR, respiratory rate; VT, tidal volume; WF, weaning failure.

**Table 2:** Demographic and clinical characteristics of the patients against the outcome.

Patient characteristics and outcomes				
	Extubation success	Extubation failure	Weaning failure	*P* value
Demographics				
Age	58 ± 16	61 ± 16	57 ± 15	0.509
Gender (male)	47 (59)	23 (68)	29 (62)	0.715
Lung status				
Normal lung	30 (61)	8 (16)	11 (23)	0.295
Airway	22 (40)	13 (24)	20 (36)	0.289
Parenchymal	27 (48)	13 (23)	16 (29)	0.294
RSBI 120				
All patients	64 ± 5.41	75 ± 3.28	80 ± 8.21	≤0.001
Normal lung	64 ± 5.4	75 ± 3.3	80 ± 8.2	≤0.001
Airway	65 ± 6.8	74 ± 3.3	86 ± 11.5	≤0.001
Parenchymal	61 ± 8.2	72 ± 3.8	83 ± 7.6	≤0.001
RSBI 5-120				
All patients	20.5 ± 1.6	29.3 ± 6.7	36.4 ± 9.3	≤0.001
Normal lung	21 ± 1.6	29 ± 6.7	36 ± 9.3	≤0.001
Airway	23 ± 5.6	33 ± 8.2	39 ± 12.1	≤0.001
Parenchymal	20 ± 2.8	34 ± 9.4	39 ± 9.0	≤0.001

## Data Availability

The data used in this study are available on request from the corresponding author.

## References

[1] Manthous C. (2000). Summarizing the logistics of liberation from mechanical ventilation. *Respiratory Care Clinics of North America*.

[2] Tobin M. J., Yang K. (1990). Weaning from mechanical ventilation. *Critical Care Clinics*.

[3] MacIntyre N. R. (2012). Evidence-based assessments in the ventilator discontinuation process. *Respiratory Care*.

[4] Frutos-Vivar F., Ferguson N. D., Esteban A. (2006). Risk factors for extubation failure in patients following a successful spontaneous breathing trial. *Chest*.

[5] Levine S., Nguyen T., Taylor N. (2008). Rapid disuse atrophy of diaphragm fibers in mechanically ventilated humans. *New England Journal of Medicine*.

[6] Kollef M. H., Silver P., Murphy D. M., Trovillion E. (1995). The effect of late-onset ventilator-associated pneumonia in determining patient mortality. *Chest*.

[7] Lloyd G. G. (1993). Psychological problems and the intensive care unit. *BMJ*.

[8] Pochard F., Lanore J. J., Bellivier F. (1995). Subjective psychological status of severely ill patients discharged from mechanical ventilation. *Clinical Intensive Care*.

[9] Esteban A., Alia I., Ibañez J., Benito S., Tobin M. J. (1994). Modes of mechanical ventilation and weaning. *Chest*.

[10] Ely E. W., Baker A. M., Dunagan D. P. (1996). Effect on the duration of mechanical ventilation of identifying patients capable of breathing spontaneously. *New England Journal of Medicine*.

[11] MacIntyre N. R., Cook D. J., Ely E. W. (2001). Evidence-based guidelines for weaning and discontinuing ventilatory support: a collective task force facilitated by the American College of chest physicians; the American association for respiratory care; and the American College of critical care medicine. *Chest*.

[12] Schoser B., Fong E., Geberhiwot T. (2017). Maximum inspiratory pressure as a clinically meaningful trial endpoint for neuromuscular diseases: a comprehensive review of the literature. *Orphanet journal of rare diseases*.

[13] Nemer S. N., Barbas C. S. V., Caldeira J. B. (2009). A new integrative weaning index of discontinuation from mechanical ventilation. *Critical Care*.

[14] Azeredo L. M., Nemer S. N., Barbas C. S. (2017). The integrative weaning index in elderly ICU subjects. *Respiratory Care*.

[15] Mabrouk A. A., Mansour O. F., El-Aziz A. A. A., Elhabashy M. M., Alasdoudy A. A. (2015). Evaluation of some predictors for successful weaning from mechanical ventilation. *Egyptian Journal of Chest Diseases and Tuberculosis*.

[16] Yang K. L., Tobin M. J. (1991). A prospective study of indexes predicting the outcome of trials of weaning from mechanical ventilation. *New England Journal of Medicine*.

[17] Segal L. N., Fiel S. B., Ruggiero S., Scoopo F., Oei E. (2005). Use of the rate of change of the RSBI during spontaneous breathing trial as an accurate predictor of weaning outcome. *Critical Care Medicine*.

[18] Deye N., Lellouche F., Maggiore S. M. (2012). The semi-seated position slightly reduces the effort to breathe during difficult weaning. *Intensive Care Medicine*.

[19] Lomholt N. (1992). A device for measuring the lateral wall cuff pressure of endotracheal tubes. *Acta Anaesthesiologica Scandinavica*.

[20] Endotracheal D. C. G. (1990). And tracheotomy tube cuff design: influence on tracheal damage. *Critical Care Update*.

[21] Liu L., Liu H., Yang Y. (2012). Neuroventilatory efficiency and extubation readiness in critically ill patients. *Critical Care*.

[22] MacIntyre N. R. (2013). The ventilator discontinuation process: an expanding evidence BaseDiscussion. *Respiratory Care*.

[23] Gnanapandithan K., Agarwal R., Aggarwal A. N., Gupta D. (2011). Weaning by gradual pressure support (PS) reduction without an initial spontaneous breathing trial (SBT) versus PS-supported SBT: a pilot study. *Revista Portuguesa de Pneumologia*.

[24] Menon N., Joffe A. M., Deem S. (2012). Occurrence and complications of tracheal reintubation in critically ill adults. *Respiratory Care*.

[25] Liu Y., Wei L.-Q., Li G.-Q. (2010). A decision-tree model for predicting extubation outcome in elderly patients after a successful spontaneous breathing trial. *Anesthesia & Analgesia*.

[26] Lessa F. A. M., Paes C. D., Tonella R. M., Araújo S. (2010). Comparação do índice de respiração rápida e superficial (IRRS) calculado de forma direta e indireta no pós-operatório de cirurgia cardíaca. *Brazilian Journal of Physical Therapy*.

[27] Epstein S. K. (1995). Etiology of extubation failure and the predictive value of the rapid shallow breathing index. *American Journal of Respiratory and Critical Care Medicine*.

[28] Shingala H. B., Abouzgheib W. B., Darrouj J., Pratter M. R. (2009). Comparison of rapid shallow breathing index measured on pressure support ventilation and spontaneous breathing trial to predict weaning from mechanical ventilation. *Chest*.

[29] Arabi Y., Karthika M., Al Enezi F., Pillai L. (2016). Rapid shallow breathing index. *Annals of Thoracic Medicine*.

[30] Chatila W., Jacob B., Guaglionone D., Manthous C. A. (1996). The unassisted respiratory rate-tidal volume ratio accurately predicts weaning outcome. *The American Journal of Medicine*.

[31] Krieger B. P., Isber J., Breitenbucher A., Throop G., Ershowsky P. (1997). Serial measurements of the rapid-shallow-breathing index as a predictor of weaning outcome in elderly medical patients. *Chest*.

[32] Kuo P.-H., Kuo S.-H., Yang P.-C., Wu H.-D., Lu B.-Y., Chen M.-T. (2006). Predictive value of rapid shallow breathing index measured at initiation and termination of a 2-hour spontaneous breathing trial for weaning outcome in ICU patients. *Journal of the Formosan Medical Association*.

[33] Meade M., Guyatt G., Cook D. (2001). Predicting success in weaning from mechanical ventilation. *Chest*.

[34] El-Khatib M. F., Zeineldine S. M., Jamaleddine G. W. (2007). Effect of pressure support ventilation and positive end expiratory pressure on the rapid shallow breathing index in intensive care unit patients. *Intensive Care Medicine*.

[35] Zhang B., Qin Y.-Z. (2014). Comparison of pressure support ventilation and T-piece in determining rapid shallow breathing index in spontaneous breathing trials. *The American Journal of the Medical Sciences*.

[36] Danaga A. R., Gut A. L., Antunes L. C. D. O. (2009). Avaliação do desempenho diagnóstico e do valor de corte para o índice de respiração rápida e superficial na predição do insucesso da extubação. *Jornal Brasileiro de Pneumologia*.

[37] Patel K. N., Ganatra K. D., Bates J. H., Young M. P. (2009). Variation in the rapid shallow breathing index associated with common measurement techniques and conditions. *Respiratory Care*.

[38] Segal L. N., Oei E., Oppenheimer B. W. (2009). Evolution of pattern of breathing during a spontaneous breathing trial predicts successful extubation. *Intensive Care Medicine*.

[39] Tobin M. J. (2001). Advances in mechanical ventilation. *New England Journal of Medicine*.

[40] Eskandar N., Apostolakos M. J. (2007). Weaning from mechanical ventilation. *Critical Care Clinics*.

